# Swallowing the Future of Cancer Prevention: Could EsoGuard Revolutionize the Fight Against Esophageal Cancer?

**DOI:** 10.1016/j.gastha.2026.101029

**Published:** 2026-05-27

**Authors:** William Ott

**Affiliations:** Rutgers Health/RWJBH, Jersey City Medical Center, Jersey, New Jersey

In a league of its own, unfortunately for decades, esophageal adenocarcinoma (EAC) has remained one of the most lethal and rapidly increasing malignancies in the Western world. The prognosis remains grim, with a 5-year survival rate often lingering just above 20 percent due to many cases being diagnosed at symptomatic stages and, hence, quite advanced.^1^ The harsh but clinical reality is that by the time a patient experiences dysphagia, the opportunity for a cure has become profoundly diminished. This diagnostic and therapeutic gap is what seems to have driven PAVmed Inc, and its subsidiary Lucid Diagnostics, to bring to market and commercialize a device that aims to shift this drastic paradigm from late-stage detection to early-stage *prevention*.^2^

The story of Lucid Diagnostics is inextricably linked to PAVmed, its parent company, which was founded in 2014 by 2 cardiothoracic surgeons and a medical device executive.^3^ The same trio who initially founded Vortex Medical Inc in 2008, also developed and eventually sold the device known as the AngioVac.^4^ Still at the helm—and seemingly the face of the company—is its cofounder, Dr Lishan Aklog. A former Harvard-trained cardiothoracic surgeon, his transition from the operating room to leading a medical device and diagnostics company was fueled by the recognition that many devastating outcomes in medicine tend to be the result of missed opportunities for early intervention. In the case of the esophagus, that “missed opportunity” is Barrett esophagus (BE)—the only known precursor to EAC.

The incidence of EAC has increased nearly seven-fold over the last 40 years.^5^ Now, let that sink in. While gastroesophageal reflux disease is the primary driver, the risk is not distributed equally. Certain high-risk profiles are well established as being linked to greater associated risk with developing EAC.^6^ Despite the latter, fewer than 10 percent of at-risk patients currently undergo the gold standard of screening—the upper endoscopy (esophagogastroduodenoscopy [EGD]).^7^ The inherent friction of the EGD—inevitable operator variability, invasive instrumentation, systemic sedation, and logistical burdens—has rendered this gold standard practically inaccessible for population-level screening.

Lucid Diagnostics believe they hold the answer to this widespread screening deficit. They seem confident they can permanently alter the landscape of BE and EAC screening but want to take it 1 step further—*prevention*. Their EsoGuard Esophageal DNA test, paired with the EsoCheck collection device, represents a seismic and technological pivot. Unlike a traditional biopsy—which requires a specialist, sedation, and a procedural suite—EsoCheck is a noninvasive, minutes long outpatient procedure. The patient swallows a vitamin-sized capsule containing a small inflatable balloon which is tethered to a thin and flexible catheter. Once inflated in the stomach, the balloon is pulled back, gently brushing the distal esophagus to collect cells. It then deflates back into the capsule to protect the sample from contamination by the upper throat or mouth.

What makes EsoGuard unique is its ability to detect metaplasia—the earliest cellular changes associated with BE.^8^ The EsoGuard Esophageal DNA test specifically targets the Vimentin and Cyclin A1 genes which serve as highly sensitive biomarkers for glandular metaplasia. In healthy esophageal tissue these genes remain unmethylated, but they undergo predictable chemical modification—the addition of methyl groups to cytosine bases—as cells transform into a precancerous state. To analyze these changes, DNA extracted from cells collected via the EsoCheck balloon undergoes bisulfite conversion, a process that converts unmethylated cytosine into uracil while leaving methylated cytosine intact. The laboratory then employs next-generation sequencing to examine 31 specific genomic locations (Cytosine-phosphate-Guanine sites) within these genes which utilizes a proprietary algorithm to provide a binary “positive” or “negative” result based on the density of methylation detected.^9^ This molecular approach effectively eliminates the subjectivity and operator variability inherent in traditional visual biopsies. By identifying these changes before they progress to dysplasia or full-blown adenocarcinoma, EsoGuard functions as a tool that can both screen and prevent. This, most importantly, becomes a preventative tool that can *complement* the undeniable and ultimately necessary, EGD.

This complementary role is supported by a strengthening foundation of clinical evidence, though not without the healthy skepticism inherent in the medical sciences. Several peer-reviewed studies, including those published in *Clinical Gastroenterology and Hepatology*, have shown EsoGuard to have a sensitivity of over 90% for detecting BE and EAC.^9^^,^^10^ Additionally, recent data from the EsoGuard BE-1 and BE-2 trials demonstrated a negative predictive value of nearly 99%, suggesting that a negative test is an extremely reliable indicator that no precancer is present—potentially sparing millions of patients from unnecessary endoscopies.

While the test has a fantastic negative predictive value, critics and some medical societies have pointed to the positive predictive value (PPV), which has hovered around 30 percent in some screening populations.^10^ A lower PPV means a significant number of patients who test positive may not actually have BE upon follow-up endoscopy. As one would imagine, this could lead to not only unnecessary patient anxiety but equally as importantly, unnecessary endoscopies for individuals who never had BE to begin with. However, many argue that in a highly lethal disease, possessing high sensitivity (not missing cases) is the more critical metric. Keeping in mind one of the fundamental principles of Bayesian statistics in medicine, utilizing pretest probability, and working to assess high-risk patient populations—the PPV could further be boosted. The ultimate cost-benefit question remains though as follows: for every 10 positive EsoGuard results, do the cost, patient anxiety, and possible risks associated with those 7 unnecessary EGDs outweigh the 3 patients whose likely otherwise fatal cancer was caught early? How about in the context of saving those 3 patients many quality years of life, or what about hundreds of thousands of dollars, if not more, by both them and the greater health system?

While the answer to that question is undeniably a difficult one, the medical establishment is beginning to take notice that change is needed. The American College of Gastroenterology and the American Gastroenterological Association have updated their clinical guidelines to reflect the viability of nonendoscopic screening. For the first time, these organizations acknowledge that “cell collection devices” combined with biomarkers are acceptable alternatives for patients who are unable or unwilling to undergo traditional endoscopy.^6^ Furthermore, the National Institutes of Health recently awarded an $8 million R01 grant to evaluate the test’s efficacy in asymptomatic at-risk individuals. The clinical study is designed to run through 2030 (launched in February 2025) and is being led by a consortium of top-tier academic centers such as Johns Hopkins University, Cleveland Clinic, University Hospitals, and none other than Case Western Reserve University—from which the technology and device were initially pioneered.^11^

The truth is that the future of EsoGuard hinges not only on ongoing clinical validation but also insurance reimbursement—namely, Medicare. It is well known the influence that the Centers for Medicare and Medicaid Services wields on commercial payors and the national benchmarks they work to devise and implement. Not to mention, many patients that are most at risk for BE are those likely to be on Medicare based plans. At the time of this writing, Lucid Diagnostics is navigating the final stages of a Medicare Local Coverage Determination. On September 4, 2025, a multijurisdictional Contractor Advisory Committee met to review the evidence on EsoGuard thus far. The meeting included experts from several major Medicare Administrative Contractors including Palmetto GBA.^12^ Ultimately, it appeared the expert consensus was overwhelmingly supportive of Medicare coverage. Unfortunately, without insurance coverage, the current out-of-pocket price for the test is approximately $1995 to $2500.^13^^,^^14^

The upfront cost of EsoGuard screening though represents a diagnostic expenditure that contrasts sharply with the economic burden of reactive EAC management. For example, surgical interventions like esophagectomies and advanced immunotherapies often far exceed $150,000 per patient.^15^ While a standard-of-care coverage model for EsoGuard requires an initial investment from payors, it serves as a high-yield fiscal hedge by avoiding what is sometimes viewed as the systemic financial toxicity of late-stage disease. This includes not only direct treatment costs but also significant intensive care utilization and long-term disability coupled with loss of productivity of those afflicted. Ultimately, transitioning from a paradigm of expensive late-stage intervention to cost-effective molecular screening could significantly reduce the total cost of care for high-risk gastroesophageal reflux disease populations globally.^16^

What can be seen as a promising development in the interim is that in January 2026, Lucid Diagnostics was awarded a national contract by the US Department of Veterans Affairs to supply its EsoGuard Esophageal DNA test through the Federal Supply Schedule. This agreement allows over 9 million enrolled veterans across the nation’s largest integrated health-care system to access esophageal precancer testing at prenegotiated pricing that matches current Medicare reimbursement rates.^17^

While the scale of this agreement is impressive, the human intentionality behind it is even more compelling. In November 2025, I had the privilege of connecting with Lucid’s Chief Operating Officer, Shaun O'Neil, whose profound transparency and unwavering vision for the future of Lucid Diagnostics left a lasting impression on me. I was deeply moved by his team’s palpable dedication and commitment to scaling their technology, as evidenced by high-level strategic presences at major clinical forums such as the MedTech Conference and American College of Gastroenterology 2025. He also described to me his experiences with systemic clinical integration as seen in their aggressive pursuit of large-scale partnerships from institutions like the Hoag Health System, commercial insurance coverage from Highmark Blue Cross Blue Shield, and additional partnerships that seemed to be imminent. Furthermore, their direct-to-consumer approaches such as their “Check Your Food Tube” mobile events are unique ways they have found to bypass slow-moving health systems and go straight to the patient. O’Neil went on to emphasize the scalability of their proprietary satellite collection model, where trained personnel (mobile nursing teams) consistently achieve sub–two-minute collection times—a critical factor for high-volume throughput in community and occupational health settings.

Their singular, noble objective became clear to me that day—to revolutionize esophageal cancer screening and significantly reduce the tragic toll of cancer-related deaths. Just as the Cologuard test revolutionized colon cancer screening by providing a home-based alternative, EsoGuard has the potential to become the standard of care for the esophagus. As a practicing Hospitalist within New Jersey’s largest academic health-care system, I have seen first-hand the destruction a diagnosis such as esophageal cancer can have on both a patient and their family’s life. For me, this is not just a test—because for many, it will be *the difference* between a routine follow-up and a possibly life shattering, terminal diagnosis.
